# The Anatomy of Teleneurosurgery in China

**DOI:** 10.1155/2011/353405

**Published:** 2011-09-20

**Authors:** Xiaohong Gao

**Affiliations:** School of Engineering and Information Sciences, Middlesex University, London NW4 4BT, UK

## Abstract

With its huge population and vast territory, China faces a great challenge in providing modern advanced health care services to all parts of the country. The advances of information communication technologies (ICTs) and the advent of internet have revolutionised the means in the delivery of healthcare via telemedicine to remote and underserved populations, which to a certain extent has been very well exploited in China, especially where 70% peasants residing in the rural areas. This paper reviews the latest development in telemedicine infrastructure in China with the focus on the development of teleneurosurgery, drawing from the results gained from a 3-year networking project between Europe and China on telemedicine (TIME, 2005–2007) funded by European Commission under Asia ICT programme, with an aim to shape up envisages of future medical care in China. Comparison with its counterparts in Europe is also addressed.

## 1. Introduction

Over the last twenty years, China has achieved unprecedented economic growth, with an accompanying growth of large numbers of wealthy and middle classes, which has led to the requirement of the building of a well-off society in a comprehensive way. To this end, China is currently in the process of reforming its health care system by equipping its hospitals with many modern medicine systems, specifically, medical imaging facilities, as well as building their own [1]. Because of the size of its territory and the number of its population as well as the uneven development of economy across the country, the distribution of the modern medical facilities allocates mainly to large cities, such as Beijing and Shanghai. In order to reach those remote regions, China has begun the development of telemedicine techniques in the late 1980s [2]. In its first attempt taking place in the first decade (1990–2000), the main focus resides on the implementation of communication networks with a faster and wider bandwidth, such as ISDN (Integrated Services Digital Network), in the hope to connect far and wide. As a direct result, built on this digital network service, *Teleeducation*, *Teleconferencing*, and *Teleconsultation* have flourished albeit mainly as a show case between a number of leading institutes [3, 4]. With the advent of World Wide Web, many internet-based services are available and more importantly are free, such as Skype, making the services of teleconferencing/consultation not only affordable but also flexible and portable, that is, a network connection being able to set up in an operation room instead of confining to a conference room, bringing visions of practical applications, such as telesurgery into a reality. Subsequently, the first case of teleneurosurgery took place in 2005 between Beijing and Yan'an with a distance of around 1300 kilometres, performing an operation of tumour removal via a keyhole [5].

By contrast, Europe has well advanced in many of these fields [6]. Firstly, Europe originated imaging field when the Nobel laureate, physicist Wilhelm Roentgen, discovered X-rays that eventually led to the birth of radiology, and thereafter the medical imaging industry. With the application of advanced computer techniques since 1970, computerised tomography (CT) and magnetic resonance imaging (MRI) were invented, leading to two more awards of Nobel Prize winners shared between the UK and the USA. With around 80,000 2D images (e.g., in Geneva Hospital) generated per day, Picture Archiving and Communications Systems (PACSs) have been developed to manage them in the mid-1980s [7]. By 2005, most European countries have installed PACS in their hospitals with Norway topping the chart with 100% hospitals. Elsewhere more than 70% hospitals in the countries of United Kingdom, Germany, and Italy are equipped with PACS. On the other hand, by 2005, PACS started to be appreciated in China and to be installed in full version among many leading hospitals. Before that, only mini-PACS (a standalone version) had been installed.

## 2. Telemedicine in Europe

To a certain extent, Europe is also leading the way in ICT. In December 1990, British physicist Tim Berners-Lee created World Wide Web while working at CERN in Switzerland, allowing computers to talk to each other through a new language HTML (Hyper-Text Markup Language), which has heralded a new era and changed the way people live forever. 

In addition, in 2009, Charles Kao, aka the *Godfather of Broadband*, was awarded half of Nobel Prize in physics [8] for his groundbreaking achievements concerning the transmission of light in fibres for optical communication while working at the UK, revolutionised the telecommunications industry, and paved the way to the advent of today's *information age*. As of 2010, submarine cables that are laid beneath the sea have linked all the world's continents [9], connecting the world into one.

In terms of the application of ICT to medicine, Europe has been very actively involved. Although the following accounts are neither comprehensive nor conclusive, it is in the intention to be representative. 

In *United Kingdom*, the National Health Service (NHS) has established a unique national healthcare system providing *free health for all *since 1948 [10]. However, at the advent of the new century, thanks to the accumulated funding constraints and the increased aging populations, the NHS faced long patient and operation waiting lists, shortages in hospital beds and community care, and inadequate medical facilities in intensive care and emergency units as well as nursing staff. This fundamental change has led to the development of the UK's largest e-health project, NHS Direct, which has been in operation since 2000. It is a 24-hour nurse-led telephone help line supported by a website, http://www.nhsdirect.nhs.uk/, covering England and Wales. A separate service, NHS24, http://www.nhs24.com/, serves Scotland. It currently handles more than half a million telephone calls per day with over 3 million web transactions each month. Because of the huge impact it has made to society, NHS Direct has been acclaimed as *the largest and most successful healthcare provider of its kind, anywhere in the world* [11]. 

In the event of collaboration with Asia on telemedicine, two European projects, TIME and WIDTH, have been/are being conducted coordinated by the author at the UK. The project TIME (*Teleimaging in Medicine*—*a Cyber bridge interfaces China with Europe on Collaborative Health Care*) (http://www.mitime.org/time/index.htm) was funded by EC under Asia ICT programme between 2005 and 2007 and has borne fruit in a number of publications [12–15]. As a follow-up project, WIDTH (*Warehousing Images in the Digital Hospital: interpretation, infrastructure, and integration*) is funded by EC FP7 under People Marie Curie programme to start in May 2011. WIDTH has 6 European partners and 5 Chinese including from the UK, Switzerland, Germany, Greece, Norway, Italy, and China.

On the other hand, in *Switzerland*, in 2001, the University Hospitals of Geneva in Switzerland and French-speaking countries in Africa initiated the RAFT project (Réseau en Afrique Fancophone pour la Télémédecine) by building a multinational telemedicine communication network in order to facilitate distance learning and teleconsultations through the internet-based platform [16]. By 2005, the network grid had been extended to Cameroon, Ivory Coast, Madagascar, and Djibouti (http://raft.hcuge.ch/). As a direct result, through ustilising the internet-based technologies, distance learning and teleconsultation were facilitated via various communication schemes. By far, this network has connected 18 African countries, extending its activities from French-speaking to English-speaking countries. 

In 2004, Trieste University at *Italy* hosted an international conference on *EuroPACS-2004 in the Enlarged Europe* and attracted more than 400 participants from 47 countries. Trieste University has a long history in the development of Open-PACS [17], in an attempt to initiate an open, scalable, and universal system with accompanying tools to store, exchange, and retrieve all health information. Elsewhere, the project *E-learning* at* University of Pisa* has developed a niche interactive system for uploading and consulting teaching materials that can be applied to performing self-evaluation tests and exams, targeting at medical students. Furthermore, the University of Pisa, Division of Diagnostic and Interventional Radiology (http://www.rad.unipi.it/) has cofunded ENDOCAS (Centre for Computer-Assisted Surgery) [18], with a goal of implementing an imaging-assisted surgery (IAS) systems to provide *information help*, *action help*, and *training help*, offering assistance on planning surgical intervention, integrating mechanic components of the robots, and simulating complex environment for surgical training, respectively. 

*Norway* has a long history of development of telemedicine thanks to its nature of national geography with an elongated shape, forming one of the longest and most rugged coastlines in the world. As early as in the 1990s, Norway pioneered projects with teleradiology services, which had been in great demand when it comes to consultation in the situations of emergencies, seeking for second opinion and information retrieval between hospitals and the primary health care units. By the end of 2005, nearly all the hospitals in Norway had digital X-ray scanners with RIS and PACS installed. Moreover, all the *Regional Health Network* could communicate with the National Health Network [19]. In 2006, in Norway, Trondheim successfully organised the 24th EuroPACS conference where TIME project hosted a workshop.

## 3. Hospital Infrastructure in China

In many ways, the situation in China is quite different from the other countries.  Table 1 lists a number of key demographic data in both China and the UK for the purpose of comparison. Being the most populated country in the world, China accommodates 1.33 billion people, representing 20% of the world population. However, China also has the 3rd largest area in the world, leading to the fact that the density in China is nearly as half as that in the UK with a ratio of 0.55 between the two countries, indicating that people are living far more apart in China than in the UK. However, in terms of bed numbers in the hospitals, China has half as many beds as in the UK with only 1.4 doctors allocated to per 1000 people. Whilst in the UK, 2.3 doctors are assigned for per 1000 people. With only 6.1% hospitals in China that have been equipped with PACS in comparison with more than 70% in the UK, China is far behind when it comes to the implementation of modern advanced medical equipment with a ratio of 0.087 between the two countries.

Hospitals in China are categorized into three four-level grades by the Chinese Ministry of Health who conducted the classification according to the facility, equipment, and staffing that each hospital can offer. The grades as shown in Table 2 include Grades 1, 2, and 3 with the highest grade being 3A+ that refers to be at an international leading position, whereas Grade 3A is expected to be the leaders nationally. There are only two hospitals that are classified as 3A+, which are Beijing Union Hospital and Beijing Hospital 301 of PLA. On the other hand, 772 hospitals are classified as Grade 3A. In total, as of 2008, there are 19,246 hospitals and 2 million doctors in service in China [2]. Within these hospitals, there are 2.2 million beds with 149 (1%) hospitals having beds over 800 (Grade 3A/3A+) and 1930 (12%) offering beds between 300–800 (Grade 2A/3B). 

Within Grade 3 hospitals, radiology departments are in place to be responsible for acquiring medical images from modern medical image scanners, including CT (Computerised Tomography), MR (Magnetic Resonance Imaging), and/or PET (Positron Emission Tomography) as well as from film digitisers to digitise X-ray films and many other forms of pictures/images.

**Figure 1:**
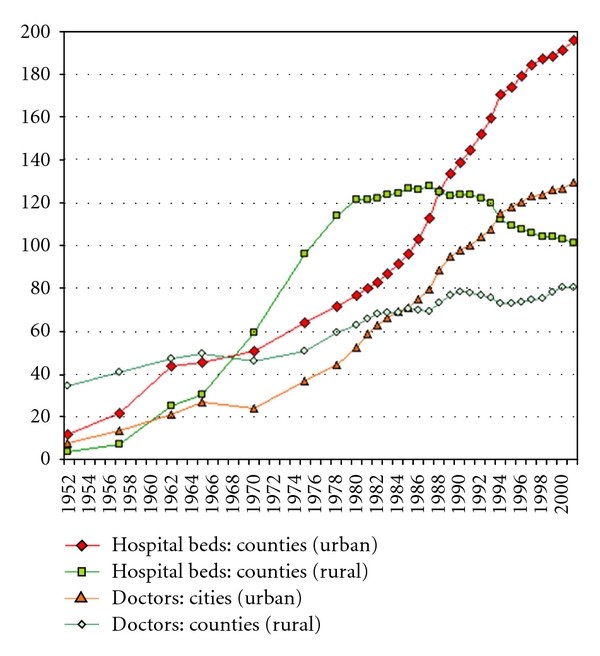
The number of hospital beds and doctors (in 10,000) in cities and countries in China between 1952 and 2002 (http://www.china-profile.com/data/fig_health_1.htm) [20].

Figure 1 displays a plot of official statistical data on the number of hospital beds and doctors distributed in both urban and rural regions from 1952 to 2000 in China [20].

It can be seen from the figure that there is a big gap between rural (green patterns) and urban (in red) regions. In terms of the number of beds, the numbers in the rural areas, in particular from the 1980s, have decreased, whereas in the cities, the number has doubled since the 1980s. On the other hand, the gap between the numbers of doctors is increasing with more and more doctors placed at city hospitals. At the meantime, the number of medical doctors and physicians in the counties stagnated or even declined since 1988.

## 4. The Infrastructure of Telecommunication in China

Telemedicine stems from technologies of communications and computer information, within which the clinical care is delivered via a line-telephone, wireless mobile phone, video-conferencing equipment, or internet between medical specialists (and patients) in two or more different locations (e.g., countries) for the purpose of conducting consulting, remote medical procedures or examinations. Hence, to provide any kind of teleservice, a telecommunication facility has to be in place first.

### 4.1. Lined Network

China has 1.33 billion people that are distributed over 22 provinces, five autonomous regions, and four metropolitan municipalities. Such a vast population and territory sets a correspondingly vast challenge to keep people in touch with each other. Therefore, starting from late 1990s, China has established more than 2 million kilometres [21] nation-wide optical cable network, built-on Asynchronous Transfer Mode (ATM), Synchronous Digital Hierarchy (SDH), and Dense Wavelength Division Multiplexing (DWDM) technologies, as well as several submarine cables, enabling communications using land-line telephones. In terms of networks that are employed for the purpose of telemedicine, there are three major routes [2], including the Golden Health Network (GHN), the international MedioNet of China (IMNC) network, and the Peoples' Liberation Army (PLA) telemedicine network. Since the implementation in 1997, IMNC has been widely utilised to telecommunications between medical specialists. Subsequently, there are around 300 hospitals (1.5%) registered on this line across China. Because it is primarily a telephone line employing a low bandwidth, the major activities between these hospitals have been limited to communications with textual data, yielding the network being analogous to an internal telephone line. 

### 4.2. Teleconferencing Systems

In order to make the presence of telemedicine felt, many hospitals in China have acquired video-based teleconferencing systems in an attempt to deliver telemedical services. Because of the high cost of proprietary video conferencing systems, the hospitals that can afford to install them in China are more likely to be the least that are in need of advice due to their own rich supply of expertise and resources. Furthermore, although China has a small number of telemedicine systems in many of these leading hospitals, they hardly communicate with each other because of different standards of hardware and software that are employed in China, yielding that these systems are essentially standalone. To access rural and regional medical centres, connections have to draw from the existing resources that are in considerably less quantity for many hospitals in the rural regions.

### 4.3. Wireless Network

Because of the wealth that China has grown since the 1980s, China has tapped into a fashion-conscious market, with special interest in those high-tech gadgets. As a result, mobile phones are a must-have accessory among young generations. Unprecedentedly, China has around 833 million mobile phone users (63%), thanks to the satellite network systems [22]. The primary service of a mobile phone is to transfer voice data, though other services have also been deployed, including email reading, web accessing, and messaging. These services however are provided at a higher cost since internet surfing involves downloading a number of pictures, taking longer to complete, in comparison with landline services. On the other hand, with such large army of mobile users, personal health systems can be exploited in the future in China based on the wireless network.

### 4.4. Internet Network

As of 2010, China has 420 million internet users (31.6% of its population) [23] with 277 million accessing web pages via cell phones. Since broadband is the most popular way to access the internet with a wired connection, with 98.1% of wired internet users choosing broadband, a total of 364 million people are now online in China, leading China to being the second largest market of internet users after USA [24] with major activities covering online chat, gaming, and internet surfing. Because of the very low cost of internet via land lines, web-based practices of telemedicine is much feasible in China, especially when many online meeting applications, such as Skype, are at the moment free available. In comparison, Europe has 475 million internet users, amounting to 58.4% of its population.

## 5. Telemedicine Infrastructure in China

The first generation of telemedicine in China started off in 1995 when a case of text-based consultation via emails had been reported, sending from Beijing to the US and later the whole world concerning a patient with heavy metal poisoning [3, 4], by which a medicine was later recommended and obtained that eventually cured the patient. Considering the high cost to setup a teleconferencing suite, internet-based applications in medicine are opted for in China in the late 1990s. It took off from a much publicised event of teleconsultation based on internet in 1998 [25], by which doctors from both China at Xi'an Medical University Hospital and the USA, Stanford University Health Care, conducted a conferring and reviewing session discussing the cases of two critically ill children, by the application of audio, video, and whiteboard, the emerging facilities at the time. Although feasible, much of the event is to prove the variability of webcast architecture, leading to a number of subsequent applications being with the same intention of demonstrations. The similar view is also shared by Hsieh et al. [26].

The real turning point of telemedical applications in China came in 2005 when telemanipulation/teleneurosurgery was conducted to remove brain tumours using image-guided keyhole surgery technique [5], after informed consent had been obtained in advance with local ethical committees. The operating distance is 1300 kilometres away in Yan'an, a mountainous region, from Beijing, with a home-made frameless stereotactic surgical robotic system CAS-BH5. The transmission of neuronavigation data, planning, monitoring, and manipulating is done through a digital network with a speed of 2 MByte/s on the platform of internet. In total, 10 patients were operated that year with 90% patients improved neurologically with no complications based on the 12-month postoperation followup. When comparing with local operations where 93.3% improvement is achieved [27], drawing from over 1000 cases, the accuracy of telesurgery is very much similar to the conventional operation although not conclusive. The following sections address this procedure in more details.

## 6. Teleneurosurgery

With the advances of information and imaging technology, application of robotic systems to the health sector is a burgeoning field in assisting surgeons manipulating, monitoring, and/or guiding operations with the advantages of being higher precision of targeting, persistence of longer duration, and the ability of preoperative planning drawing from patients' images. As of 2001, there are around 270,000 cases of image-guided robotic operations conducted per year worldwide [28].

In China, around 6.1% hospitals (Grade 3) (as opposed to 70% in the UK) are equipped with these modern imaging scanners due to its higher maintenance cost incurred on not only the hardware, but also personnel who will be able to operate the scanners, to perform some repair, and to write some computer code if necessary. Therefore sharing both these equipment and expertise are in greater need. Teleneurosurgery is one of the applications in terms of sharing techniques on medical images, telecommunications, and robots.

Unlike keyhole approaches applied on many other organs (e.g., in the abdomen), where a microcamera can be inserted to provide an augmented view, for a brain which is a compact organ and extremely high value, there is simply no “room” to accommodate any extra instrument as every tissue in a brain plays an important role to ascertain a person's normal life. Minimal invasion and sacrifice of healthy tissues are hence the prerequisite for a brain intervention. Therefore, it took much longer in the domain of brain to have a keyhole surgery than in many other domains. The first case of image-guided neurosurgery took place in 1985 [29] in the USA for the procedure of biopsy using the robotic stereotactic technique, that is, to probe the tumour with a tip through a small burr hole drilled on the skill. In order to “see” inside a brain while performing the keyhole surgery, by which a needle/catheter was introduced through the hole to extract tissues, surgeons relied on CT images that performed scanning intraoperatively where the robotic system was mounted on the gantry of the CT. Although this system was applied only for biopsy at the time, it has potentials to progress further. Because it is a robotic system, that is, controlled by a computer, it provides the possibility of being carried out “remotely”, giving rise to the name of telerobotic system when coupled with the advances of computer technique.

### 6.1. Frame and Frameless Stereotactic

Since a brain resides inside a skull, many approaches have to be developed in order to *see* and *locate* the exact position in a brain. Stereotaxis is the most popular one and was conceived by Horsley and Clarke [30]. The method employed an external, three-dimensional frame of reference to locate a position in the brain and was utilised on animals as early as in the 20th century [31]. The frame is a mechanical apparatus attached to an animal's head and incorporates a Cartesian coordinate system. In this way, the movement of a probe to be inserted into the brain can be monitored. However, the variations between the landmarks on a skull and the targets in the brain vary considerably from person to person and from time to time even for the same person, the application of stereotactic device to humans only began in the 1950s [32]. Since then, a number of stereotactic frames have been developed worldwide that can be adjusted manually. 

**Figure 2:**
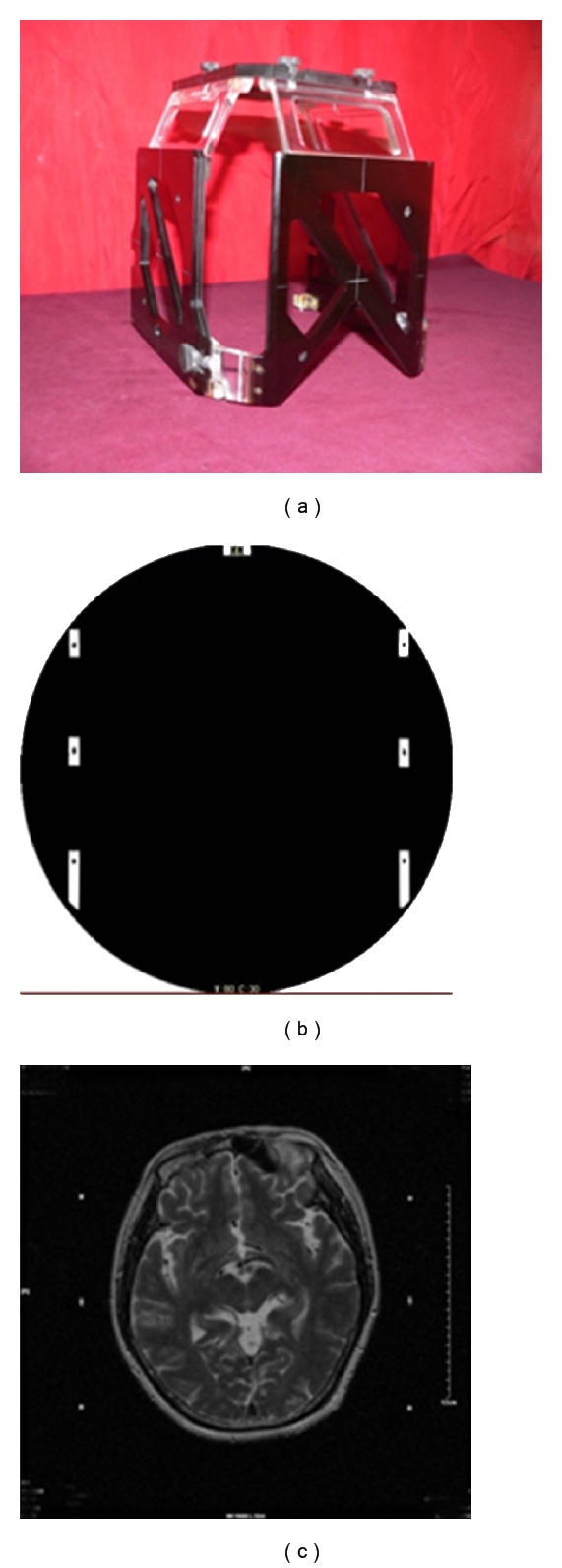
(a) A head frame with contrast agent of copper sulphate solution filled in the N shape frames. (b) A CT slice with the frame on. (c) An MR slice at the same head position (same z direction) as that in the CT.

Figure 2(a) illustrates a frame with fiducial markers, which is applied in the Navy General Hospital in China. To increase the contrast of frame landmarks while being scanned using either CT or MR, contrast agent of copper sulphate solution is usually filled in the frames of N shapes. In Figures 2(b) and 2(c), the positions on those N shape frames are shown on a CT and an MR images (the dots) for the same patient and at the same head position, which will be referenced for the procedure of registration in a later stage.

To apply such a frame in a surgery, two physical spaces have to be registered together to ensure the coordinate systems can be transformed from one to another. One space is for the frame (or head space), whereas another is for the brain (brain space). Since the brain space is unseen, brain atlases have therefore been developed to help navigating inside a brain. Because of the variations in brain size, shape, and status of diseases, a position based on a brain atlas of “one size” tends to give considerably deviations from the target, leading to a declined application of such technique to neurosurgery. 

The advent of imaging techniques, especially CT and MR, in the early 1980s, has renewed the use of stereotactic apparatus into neurosurgery [33], where the brain atlases can be entailed by a patient's brain images, leading to a patient-specific registration of the brain targets. 

In the event of registration, the calculations involve the mapping between a patient's head (frame space), brain, and images (image space), which are achieved with the help of landmarks as shown in Figure 2, yielding the development of many accurate approaches of registrations, taking forms of linear, affine, and deformable. 

**Figure 3:**
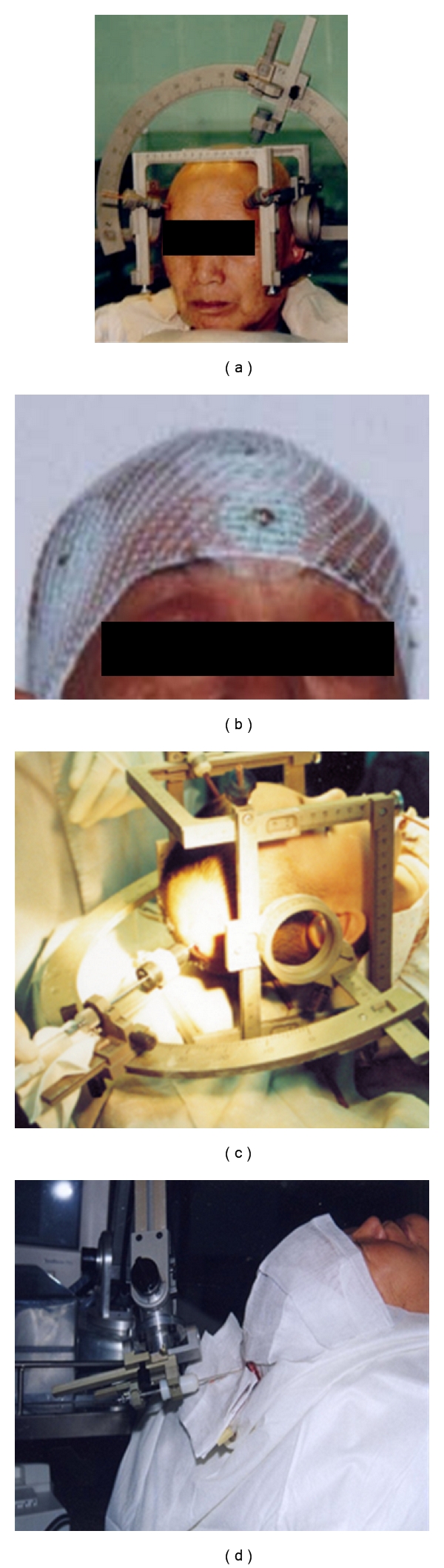
The comparison between frame (a, c) and frameless with markers (b, d) in the operation.

On the other hand, wearing a frame is cumbersome in both a scanning suite for acquisition of images and an operation room, while, in the process of a surgery, frameless stereotaxis has gradually replaced the frame. In this way, landmarks, that is, fiducial marks, on a skull have to be defined in advance to assist geometric registrations. Significantly, with the benefit of advances of computer hardware and software, frameless stereotactic can achieve the same precision as that with a frame. In addition, the visualization of 3D volumetric image data preoperatively obtained offers surgeons with surgical planning, tumour boundary delineation, and optimised route to minimise the risk to healthy tissues in the brain.  Figure 3 demonstrates the methods using both frame (a and c) and frameless stereotactic neurosurgery. In total, about 4,000 operations with frame and 1,500 with frameless stereotactic have been performed at Navy General Hospital [34] at Beijing China, led by Professor Zengmin Tian, the Vice President of NGH.

### 6.2. Robotic Systems

With the help of a frame or frameless stereotactic system, minimally invasive techniques to remove brain tumour are achievable by locating a precise position on a skull (and hence the target in the brain). The procedure normally involves the drill of a burr hole (1–10 mm), which usually being done manually. Because of the lengthy procedure of an operation, surgical dexterity can be limited to a certain extent by surgeons' physiological tremor. Therefore, robotic hands or robots have been introduced into the operation theatres in the late 1980s [35, 36], which could replace stereotactic frame and complete precise position location, and precision bone drilling, and are defined as “programmable multifunctional manipulators designed to move material, parts, tools or specialized devices through variable program motions for the performance of a variety of tasks” [37, 38]. 

In China, the application of robotic system for neurosurgery began a decade later than in Europe and the USA, which is mainly confined to tumour resection to achieve minimally invasive operations, specifically, the removal of craniopharyngioma, a benign tumour derived from pituitary gland embryonic tissue. Although, at the time, commercially, such a system was available, such as RoboDoc, the cost was beyond their considerations. Hence, from 1997 to 2007, in collaborating with Beijing University of Aeronautics and Astronautics, Navy General Hospital developed their own robotic systems from CAS-R-1 and CAS-R-2 (acronym of Computer Assistant Surgery-Robot, Type 2) to CAS-BH5 as depicted in Figure 3 (private communications). The robotic system employs a six-jointed PLC-controlled (a programmable logic controller) and motorised robotic arm (Figure 3(a)) as well as a software system that is responsible for location calculation, markers recognition, surgical planning, and intraoperative navigation.

#### 6.2.1. Hardware System

The robotic arm is designed to mimic the joints of a human arm comprising of a shoulder (joint 3), an elbow (joint 2), and a wrist (joint 1), allowing near unlimited range of angular movements and the smoothness of the motion. In addition, the robot is mounted on an external support (joint 4) incorporating a base (joint 5) that is positioned in such a way as to avoid interference with surgeons' manoeuvres. Prior to a surgery, four markers attached to a head are applied to position all joints on a trajectory to the intended targets, that is, to allow an accurate registration between a robotic arm, head, and imaging-defined targets. In this way, a specially designed probe attached to joint 6 (the tip) is able to perform the tasks of biopsy or tumour resection (red circle in Figure 3(c)). On the other hand, to create a skull opening, a power twist drill is passed through the bushing of the probe holder on joint 6 where skull perforation is performed in the exact trajectory to be followed by the probe. In principle, the probe holder can be positioned at any distance from the skull. 

#### 6.2.2. Robotic Surgery

As soon as the desired positioning is selected, the robotic software system calculates the distance necessary to reach the intended target within the brain. The necessary probe length can then be transmitted manually to the probe that is then introduced into the brain manually, or the probe can be fixed to the robot arm whereby the probe introduction can be carried out by the robotic motor system. In terms of the time spending on positing a robot, by using a PLC-controlled motor, it takes only a few seconds to move a robotic arm into the intended operative field, to perform probe on the trajectory marked on the skull and to move it out of the area. Such a technique makes it easy to mark the intended site of cranial opening, move the robot out of the field, create the necessary cranial opening, and then rapidly move the robot arm back into the field. The surgeon thus has unobstructed access to the patient's head but has the ability to return the robotic arm quickly to the field as desired. The accuracy of the robotic arm in reaching the target is within the error range of ±0.32 mm between planned and actual target [39, 40].

The first case of operation on tumour removal was carried out in China in 1997 [5] with a custom-made robot, CAS-R-2. In the following 10 years, over 2000 cases of operations have been carried out by it with total effect rate of 93.3% [39]. The advantages include higher accuracy of targeting, being able to conduct presurgical planning, and synchronised movement between a patient's head, images, and robotic hand (robot space).  Figure 5 illustrates part of tumour that has been dissected as intended using the stereotactic robotic technique. At Figures 5(a), 5(b), and 5(c) (T1 MR), a tumour occurring in the central part of the brain on different brain locations (pointed by arrows) is shown, whereas the bottom row (T2 MR) gives detailed information to depict the partial removal of the tumour.

### 6.3. Telemanipulation and Telemonitoring

Significantly, the advent of robotics in an operating room provides the ability to position the probe-directing system rapidly, to compute the necessary trajectory and target coordinates in an error-free manner, and to remove tumour more completely or as intended. This has led to the possibility of a robot being controlled remotely, that is, to carry out teleneurosurgery.

**Figure 4:**
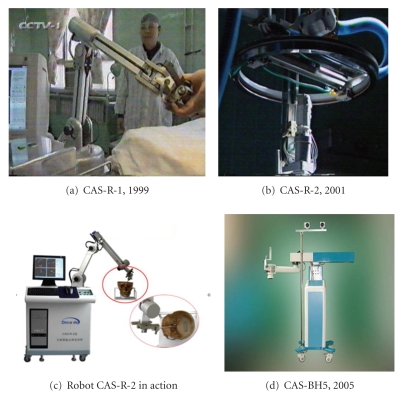
The time line of the development of home-made robotic system for neurosurgery in China from Generation 1 to Generation 5 (private communication).

As a result, in 2005, the CAS-R-2 system was modified into CAS-R-BH5 (Figure 4(d)) that was employed in performing the first series of teleneurosurgery in China, exclusively for removal of tumours and taking place between Beijing where Navy General Hospital resides and Yan'an, a mountainous city, with the distance of 1300 km in between.

With five degrees of freedom (that is, 6 joints) and dimensions of 280 × 800 × 1100 mm^3^, CAS-R-BH5 weighs about 40 kg and comprises a robot hand and two sensors that register the robotic space with head space as well as brain MR images. The system runs three modules that are responsible for conducting surgical planning, target directing, and telemanipulation operations, respectively.

**Module 1**

The system of surgical planning offers surgeons with expedient tools to store, retrieve, and analyse relevant data, to study collectively the case history, to visualise reconstructed 3D images in order to define the boundary of a target for more completed removal, and to locate contrally the brain tumours by the safest and least invasive route possible.

**Module 2**

Subsequently, the module of target directing is concerned with robotic arm in arriving at the precise position of the skull to perform the opening and pointing to the direction of the route defined by Module 1. Furthermore, via a rapid and accurate measurement and calculation, the length of the probe (catheter) is determined to minimise the trauma to normal tissues.

**Module 3**

In light of communications, Module 3 focuses on telemanipulation systems, spanning from network communication, video transmission, and graphics simulation to human-machine interaction, providing technical supports for surgeons who are performing the teleoperation.

The teleneurosurgery system is composed of two computer terminals, one being the master to conduct the remote guiding and controlling and another being the operation terminal residing at the same room as the patient, to be applied to conduct the resection of physical tumour.  Figure 6 schematically illustrates the relationship between the two [5].

The platform of telecommunication is the Internet with the speed of transmission being 2 Mbytes/s. Although it is lower than the current standard of broadband with a typical speed of 20 Mbyte/s, the transmission was good enough to allow real-time online visualization and communications. The following procedures are then required to complete a telesurgery successfully. Before the operation, that is, within 24 hours, the patient should have the brain images taken, which can be done by any hospital nearby that has the scanning facility.

- Slave to master: firstly, the patient's data (e.g., MR/CT scan, history record, and the live view of patient in the operation room) from slave computer are transmitted to the master one.
- Master to slave: the experts send back the surgical plan and confirmations of surgical procedures as shown in Figure 6(b), including the precise movement, and the robot arm should comply in order to arrive at the exact location predefined from Module 1 on the skull of the patent.
- Slave to master: the registration data between robotic arm and the fiducial markers on the patient's skull to ensure the error was below the predefined threshold. Otherwise, the following step is repeated.
- Master to slave: a series commands that the arm should follow in light of six joints instructed from master to slave then to the robot, for example, forward/backward, left/right, up/down, side tilting, anterior/posterior tilting of the supporting device itself, and forward/backward of the probe [5].

The speed of the movement of robotic arm was at 8 mm/s. Before the surgery, 4 markers are attached to the patient's skull, which are employed to track the movement of robot arm in relation to the brain images. After the induction of local anesthesia on the skull, the patient's head is rested stably on an operative frame (e.g., a cushion). The robot then automatically registers and verifies the markers via the visual camera and sends back to the master site with registration data to be checked.

**Figure 5:**
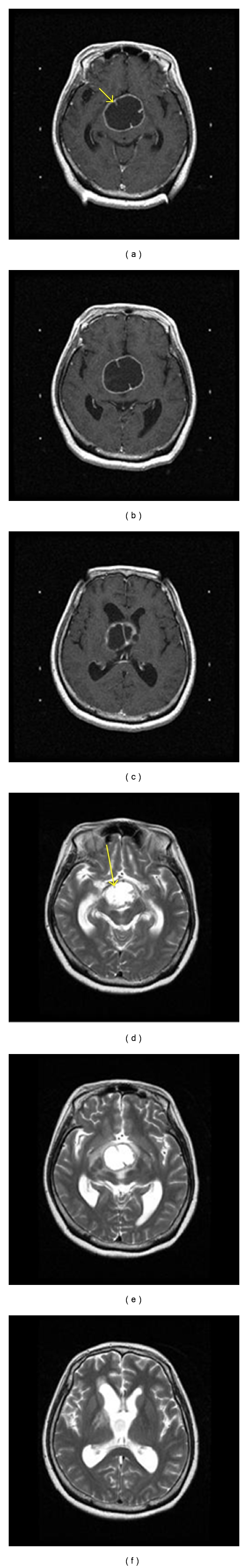
The MR scans (T1 (a, b, c) and T2 (d, e, f) illustrate the removal of part of tumour (at central position pointed by an arrow).

**Figure 6:**
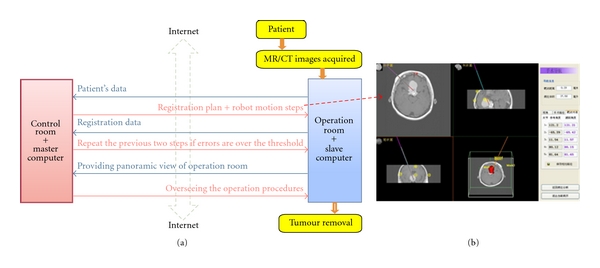
Telemanipulation between control and operation rooms (a). Planning path and robotic movements given from master room to the operation room (b).

**Tumour removal**

after the registration error is below the predefined threshold, the surgeons at the operation room take over and perform subsequent actions: incising the skin, opening a burr hole, equipping robot arm with a catheter, directing into the parenchyma, and extract tumours in the form of liquid. These surgeons in the operation room have been trained in advance with the knowledge of stereotactic neurosurgery and are capable to handle any intraoperative complications. On the other hand, the experts at the master site are monitoring the process closely online via the camera mounted in the operating room and lending a helping hand both visually and audibly. The whole process takes about 30 minutes to complete. After the surgery, the patient undergoes a postoperation CT scan of thin-sliced to ensure the tumour has been resected according to the plan by comparing with the preoperative scans as illustrated in Figure 5.

**Figure 7:**
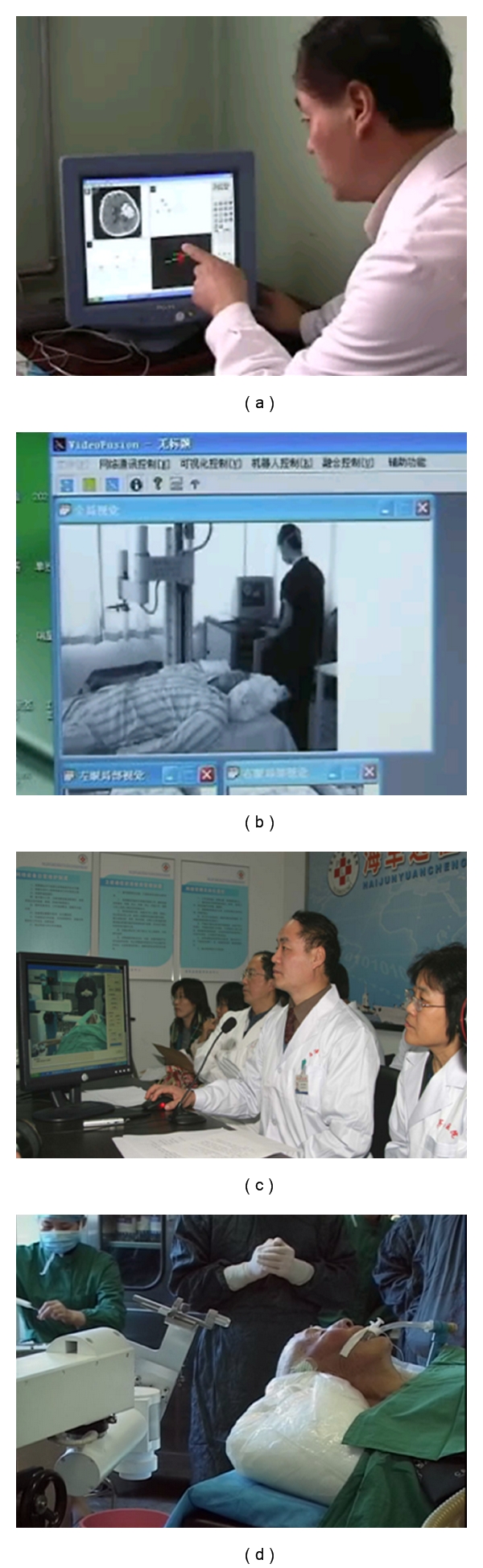
Telementoring. (a) The control room where surgical planning takes place. The person in charge is Professor Tian at Navy General Hospital at Beijing. (b) The master computer screen showing the activities in the remote operation room; (c) telementoring and telemanipulation led by Professor Tian; (d) activities in the operation room.

Figure 7 presents four screenshots showing the whole process of the teleoperation, where (a) shows the control room where surgical planning takes place; (b) shows the computer screen showing the activities in the remote operation room; (c) shows telementoring and tele-manipulation; (d) shows activities in the operation room.

The clinical outcome is then graded based on Glasgow Outcome Scale (GOS) [41], a 5-point score given to victims of traumatic brain injury at some point in their recovery. These 5 points include

1. dead,
2. vegetative State (meaning the patient is unresponsive, but alive; a “vegetable” in lay language),
3. severely Disabled (conscious but the patient requires others for daily support due to disability),
4. moderately Disabled (the patient is independent but disabled),
5. good Recovery (the patient has resumed most normal activities but may have minor residual problems).

GOS is a very general assessment of the general functioning of the person who suffers a head injury. Between 2005 and 2006, 32 patients underwent surgery of tumour removal at Yan'an with this technique of telesurgery and were all successfully recovered without any complications, the results based on the followups for the patients, which took place between 3 to 14 months after the operations with the average of 12 months. The average GOS score is 4. The mean accuracy of remote fiducial registration is within the range of 1.50 mm, and the standard deviation is 0.32 mm between the planned and actual target [39]. More specifically, in 2005, according to the results of 10 patients who were operated on using teleneurosurgery procedure, four patients recovered nearly completely with 5 points on the GOS scale, whereas three with 4 GOS scores. Two patients are with GOS score of 3 and one with 2.

## 7. Conclusion

In China, Navy General Hospital is the only one that has the skill to perform keyhole neurosurgery. Given the size of the area in China, it would be very difficult for some patients both economically and physically to go to Beijing should not for the availability of teleneurosurgery technique, especially that many of patients are young children. Likewise, due to the workload the operation team have in their own hospital, it is very impractical for them to travel around whole China to perform the operations. Therefore, in China, teleneurosurgery is one way forward towards the development of telemedicine, balancing the disparity of medical care distributions.

On the other hand, although the techniques of telemedicine/telesurgery are available, the calls for employing them vary from country to country. For example, in the UK, medical resources are evenly distributed across the whole country and are provided by the NHS, coupled with adequate emergency systems that are equipped with fast means of transportations. Furthermore, the population density in the UK is nearly double that in China. Therefore, a patient can be easily located to an intended nearby hospital within required time. In contrast, China has a very different geographic pattern. Many places, for example, mountainous regions, are difficult to locate even with several means of transportations. Significantly, China is still in the developing stage and has a long and bumpy way to go in order to allow everyone access their much needed health care, especially for those 70% peasants who live in the remote areas, far afield from those big cities where modern medical equipment are concentrated. In this case, China should be in favour of the services allowing telemedicine/teleservices to be provided over the Internet. By this way, it can save time, personnel, and cost substantially without compromising patients' safety. Considering that over 300 million people are with land telephone line and over 800 million are with mobile phones, teleservices in medicine have huge potentials in China. 

One of the promising areas is telesurgery like the one pioneered by the Navy General Hospital in Beijing. The strength of the approach lies in the knowledge transfer. Due to the limited number of experts in the field, performing remote surgery retains its positive features. In particular, it has the ability that one expert team can supervise several telesurgeries that are operated at different locations at the same time, reducing patients' waiting list, prolonged suffering, and the cost of experts' time, while maximising the usage of existing resources.

In the developing countries, telemedicine remains to be a way forward to allow health care services being accessed evenly by the people. To this end, collaboration with the developed countries is one of the future trends in order to acquire expertise, knowledge, and experience with less cost and in a short-time period.

## 8. Discussion on Chine Telemedicine

Due to the disparity of economic development in both Europe and China, China has to develop telemedicine systems that fit its purpose instead of simply copying the systems that work well in the other countries. For instance, at the UK, the success of NHS Direct owes much to the fact that everyone in the UK (and many other developed countries) is connected with 100% of households having land-line telephones and 122% people have mobiles (many users have more than one handset), which proffers advices to anyone who has any doubt on their health. Whereas in China, because of the huge number of the population, large number of knowledgeable nurses has to be required to provide the service, which is highly unlikely. In addition, many peasants who live remotely do not have any means of communications (more than 30% households without land-lined phones) nor electricity. Centralised local or regional medical centres hence remain to be the trends and can facilitate telemedicine services.

In the light of digital divide between rural areas and big cities, it is very unlikely in a short period for China to go to paperless (digital) hospital. Region-based medical centres or hospitals are still in demand to provide basic health care and advice to the local people.

The nature of relative finance independence of hospitals in China has led hospitals to be competitors as well as collaborators, hampering the development of telemedicine. A typical example appears to be in the purchase of PACS. On the one hand, the main purpose of PACS is to allow communications between each other. Whereas on the other, some hospitals install PACS with the intention to prevent the data flow going to the other sites, albeit for the benefit of security. Therefore a centralised management system with top-down architecture should be promoted to share and communicate. 

Since over 60% population own a mobile phone, wireless applications could be considered in providing a reliable, day-to-day modern medical service to any location and in any environment. This approach should be treated as a strategic means of meeting the priority of providing medical care to everyone. Furthermore, any new gadget can appeal to a huge number of followers in China, and the trend could be exploited towards the application of telemedicine.

On the other hand, with respect to telepresence in the operation room, teleneurosurgery has raised perspective on both necessity and difficulty in China, with a number of issues remaining to be further exploited. At present, due to the low bandwidth of transmission, all image data are transferred before the operation to overcome the time lag. Since the remote control room is in the position of monitoring rather than teleoperating, near real-time communication of numerical data (e.g., the angles of a robot's position and registration data) is more important. In this way, these data can be delivered over either telephone line or mobile phone should the Internet network breakdown. However, real-time overview of the operation room will certainly help to a great extent. Additionally, in this study, all the surgeons in the operations rooms have been very well trained in the control centre in advance and are capable of dealing with any situation of complications that might arise. In the future, however, to take full advantage of telepresence/telemedicine, those trainings can also be concerted via telepresence facilities to reduce the cost especially when local hospitals are underresourced substantially. Although currently all the operations are very well planned and conducted successfully thanks to the experienced surgeons at both control and operation sites, to widen the application of this technique, local level of education should be established, especially in the remit of manual handling of a drilling system in the event of possible failure of the robotic mechanical device. This again has led to the issue of training of local staff to be able to manipulate the robotic hand manually according to the preplanned path.

Although China is getting rich in a number of aspects, accessing adequate medical care is still far afield, especially for those people who live in the countryside. Telesurgery is one way towards bridging this disparity, paving the way for many other similar developments. It is anticipated that with the advances of computer information and communication technology (ICT), telemedicine not only can serve people with regards to health issues, but also can provide additional means of education, closing the gap in both literacy rate and economic development between rural and urban areas. At present, many people from China as well as from many other countries are sceptical about being treated by a robot and still prefer face-to-face consultations, which have hampered the development of telemedicine and its related applications to a certain extent. Sufficient education is therefore in need, in particular, in the field of ICT.

## Figures and Tables

**Table 1:** Infrastructure data in China with data up to 2008 in comparison with the counterparts in the UK [42].

	Population (million)	Areas (km^2^)	People/area	Hospitals	Beds/per hospital (average)	Doctors	Doctor/1000 people	Hospital with PACS
China	1,330	9,600,000	138	19,246	130	2,000,000	1.4	**6.1%**
UK	60.6	243,820	248	775	250	138,000	2.3	**70%**
Ratio	23.10	39.37	**0.55**	24.83	**0.52**	14.49	**0.60**	**0.087**

**Table 2:** The number of classified hospitals with in-patient [1, 43].

Level	3 (3A+, 3A, 3B, 3C)	2 (2A, 2B, 2C)	1 (1A, 1B, 1C)	Total
Number of hospitals with in-patient	1192	6780	4989	**12,961**
